# Comparative Genomic Analyses Reveal a Specific Mutation Pattern Between Human Coronavirus SARS-CoV-2 and Bat-CoV RaTG13

**DOI:** 10.3389/fmicb.2020.584717

**Published:** 2020-11-30

**Authors:** Longxian Lv, Gaolei Li, Jinhui Chen, Xinle Liang, Yudong Li

**Affiliations:** ^1^State Key Laboratory for Diagnosis and Treatment of Infectious Diseases, National Clinical Research Center for Infectious Diseases, Collaborative Innovation Center for Diagnosis and Treatment of Infectious Diseases, The First Affiliated Hospital, College of Medicine, Zhejiang University, Hangzhou, China; ^2^Department of Biological Engineering, School of Food Science and Biotechnology, Zhejiang Gongshang Univeristy, Hangzhou, China; ^3^College of Biological and Chemical Engineering, Jiaxing University, Jiaxing, China

**Keywords:** SARS-CoV-2, synonymous mutation, codon usage, natural selection, Bat-CoV

## Abstract

**Background:**

The outbreak of severe acute respiratory syndrome coronavirus 2 (SARS-CoV-2) in Wuhan, China, rapidly grew into a global pandemic. How SARS-CoV-2 evolved remains unclear.

**Methods:**

We performed a comprehensive analysis using the available genomes of SARS-CoV-2 and its closely related coronaviruses.

**Results:**

The ratio of nucleotide substitutions to amino acid substitutions of the spike gene (9.07) between SARS-CoV-2 WIV04 and Bat-CoV RaTG13 was markedly higher than that between other coronaviruses (range, 1.29–4.81); the ratio of non-synonymous to synonymous substitution rates (dN/dS) between SARS-CoV-2 WIV04 and Bat-CoV RaTG13 was the lowest among all the performed comparisons, suggesting evolution under stringent selective pressure. Notably, the relative proportion of the T:C transition was markedly higher between SARS-CoV-2 WIV04 and Bat-CoV RaTG13 than between other compared coronaviruses. Codon usage is similar across these coronaviruses and is unlikely to explain the increased number of synonymous mutations. Moreover, some sites of the spike protein might be subjected to positive selection.

**Conclusions:**

Our results showed an increased proportion of synonymous substitutions and the T:C transition between SARS-CoV-2 and RaTG13. Further investigation of the mutation pattern mechanism would contribute to understanding viral pathogenicity and its adaptation to hosts.

## Introduction

Severe acute respiratory syndrome coronavirus 2 (SARS-CoV-2), also known as 2019-nCoV, is a novel coronavirus (CoV) isolated from patients with pneumonia in China in 2019. SARS-CoV-2 has a similar incubation period (median, 3 days) and a relatively lower fatality when compared with SARS-CoV or MERS-CoV (Jiang et al., 2020), but the reproductive number of SARS-CoV-2 is estimated to be higher than that of SARS-CoV (Liu et al., 2020). Moreover, some laboratory-confirmed symptomatic cases lack apparent cough, fever, or radiologic manifestations, making it difficult to identify all infected patients in a timely and accurate manner (Xu et al., 2020). As of July 17, 2020, patients infected by SARS-CoV-2 have been diagnosed in more than 200 countries, and more than 13 million confirmed cases and 560,000 deaths associated with SARS-CoV-2 infection have been reported worldwide.

The genetic information of a virus is essential for its classification and traceability and its pathogenicity. At the whole genome level, the sequence identity of SARS-CoV-2 is 50% to that of MERS-CoV; 79% to that of SARS-CoV; 88% to those of two bat-derived SARS-like coronaviruses, Bat-SL-CoVZC45 and Bat-SL-CoVZXC21 (collected in 2018 in Zhoushan, China); and 96% to that of Bat-CoV RaTG13 (collected in 2013 in Yunnan, China) (Zhang L. et al., 2020; Zhou et al., 2020). Each genome of all SARS-CoV-2 strains now submitted online contains nearly 29,900 nucleotides (nt), which are predicted with at least 14 open reading frames (ORFs) (5′–3′), such as *ORF1ab* (*P*, 21,291 nt), *spike* (*S*, 3,822 nt), *ORF3a* (828 nt), *envelope* (*E*, 228 nt), *membrane* (*M*, 669 nt), *ORF8* (366 nt), and *nucleocapsid* (*N*, 1,260 nt) (Wu et al., 2020). Among them, the spike gene encodes a glycoprotein that is crucial to determine host tropism and transmission capacity and is highly divergent compared with that of Bat-CoV RaTG13 (93.1% nucleotide identity) (Lu et al., 2020; Wu et al., 2020).

Generally, the rates of nucleotide substitution of RNA viruses are faster than those of their hosts, and this rapid evolution is mainly shaped by natural selection (mostly purifying selection) (Lin et al., 2019). Genetic mutations such as nucleotide substitutions, deletions, and insertions have been frequently reported when comparing SARS-CoV-2 with other viruses (Lu et al., 2020; Wu et al., 2020; Zhou et al., 2020). In this study, we investigated the potential mutation pattern of SARS-CoV-2 by comprehensive comparative genomic analysis of non-synonymous/synonymous substitutions, relative synonymous codon usage (RSCU), and selective pressure to explore their potential roles in virus evolution.

## Materials and Methods

### Sequence Data

The SARS-CoV-2 reference genomes Wuhan-Hu-1 (NC_045512) and WIV04 (MN996528) were downloaded from the GenBank database. Twenty-one closely related coronavirus complete genome sequences and their coding sequences were also downloaded from the GenBank database (Table 1).

**TABLE 1:** Coronavirus genome sequences used in this study.

**Strain name**	**Accession number**	**Host**
WIV04	MN996528	Human
SNU01	MT039890	Human
RaTG13	MN996532	Bat
CoVZC45	MG772933	Bat
Tor2	AY274119	Human
civet007	AY572034	Civet
WIV1	KF367457	Bat
LYRa11	KF569996	Bat
HKU3-8	GQ153543	Bat
BtKY72	KY352407	Bat
Zhejiang2013	NC_025217	Bat
EMC2012	JX869059	Human
CAMEL-363	KJ713298	Camel
RSA2011	KC869678	Bat
bCoV-ENT	NC_003045	Bovine
MHV-A59	NC_001846	Mouse
HKU1	NC_006577	Bat
FarmA	MF094681	Pig
HKU2	NC_009988	Bat
229E	NC_002645	Human
PEDV	NC_003436	Pig

### Phylogenetic Analysis

Genome sequences were aligned using MUSCLE v3.8.31 (Edgar, 2004), followed by manual adjustment using BioEdit v7.2.5. Phylogenetic analyses of the complete genome were performed using the maximum-likelihood method and general time-reversible model of nucleotide substitution with gamma-distributed rates among sites (GTR + G) in RAxML v8.1.21 (Stamatakis, 2014). Support for the inferred relationships was evaluated using bootstrap analysis with 1,000 replicates, and trees were rooted using the alpha-coronavirus lineage as an outgroup.

The coding sequences were translated and aligned using the MEGA X program (Kumar et al., 2018), and then codon-based sequence alignment was used for further analysis. Phylogenetic analyses of coding sequences were performed using the MEGA X software. The changes in amino acids or nucleotides for each coding sequence were analyzed using in-house Perl scripts. Both NT and AA changes were counted by comparing to the reference strain in each CoV lineage.

### Estimation of Synonymous and Non-synonymous Substitution Rates

The number of synonymous substitutions per synonymous site (dS) and the number of non-synonymous substitutions per non-synonymous site (dN) for each coding region were calculated using the Nei–Gojobori method (Jukes–Cantor) in the PAML package.

The adaptive evolution server^1^ was used to identify the eventual sites of positive selection. For this purpose, the mixed-effects model of evolution (MEME), which allows the distribution of dN/dS (*ω*) to vary from site to site and from branch to branch at a site, was used (Murrell et al., 2012). This test allowed us to infer episodic and pervasive positive selection at individual sites.

### Synonymous Codon Usage Analysis

To investigate the potential RSCU bias of the spike protein from SARS-CoV-2 and its closely related coronaviruses, the coding sequence of the spike protein in these coronaviruses was calculated with CodonW 1.4.4^2^. The RSCU of human genes was retrieved from the Codon Usage Database^3^. The potential relationships among these sequences were calculated using a squared Euclidean distance [${\text{d}}_{i\,k}=\sum_{j=1}^{p}\left({\text{X}}_{i\,j}^{2}-{\text{X}}_{k\,j}^{2}\right)$]. In addition to RSCU, the effective number of codons (ENc) was used as a simple metric to verify codon bias and explore the source of the virus.

### Statistical Analysis

Statistical analyses were performed using the R statistical package (version 3.2.2). Chi-squared test was used to compare any two data sets, and the data were considered significantly different if the two-tailed *p* value was less than 0.05.

## Results

### The Mutation Pattern Between SARS-CoV-2 and Its Closely Related Coronaviruses

From December 2019 to February 2020, the genome sequences of 108 strains of SARS-CoV-2 virus were submitted to the global initiative on sharing avian influenza database (GISAID) worldwide. Compared with the standard SARS-CoV-2 strain WIV04, 98 point mutations were detected at 93 nucleotide sites in all SARS-CoV-2 strains with genome sequences available on February 25, 2020. However, only 58 of these nucleotide mutations caused changes in amino acids. Among them, 15 nucleotide substitutions at 14 sites caused changes in 7 amino acids of the spike protein.

**FIGURE 1:**
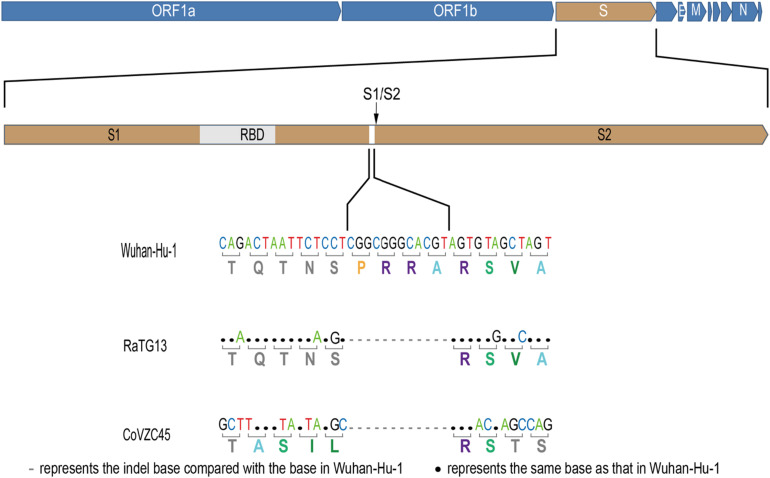
Base mutations of the largest insertion in the spike sequence of severe acute respiratory syndrome coronavirus 2 (SARS-CoV-2) against the most closely related SARS-like CoV RaTG13 and Bat-SL-CoVZC45.

The newly identified SARS-CoV-2 strain WIV04 genome sequence is closely related to Bat-CoV RaTG13 and Bat-SL-CoVZC45, which were collected from the horseshoe bat *Rhinolophus affinis* (Zhou et al., 2020). Compared with the RaTG13 genome, many nucleotide substitutions are observed, but only five small insertion and deletion (indel) mutations. The largest insert segment in the WIV04 genome was the “CGGCGGGCACGT” sequence, which is located near the boundary of the S1 and S2 regions of the spike protein. Interestingly, only synonymous mutations are observed near this insertion sequence (Figure 1). Compared with the Bat-SL-CoVZC45 genome, this insert segment is detected in the SARS-CoV-2 genome as well, but non-synonymous mutations are also observed around this insert sequence.

**FIGURE 2:**
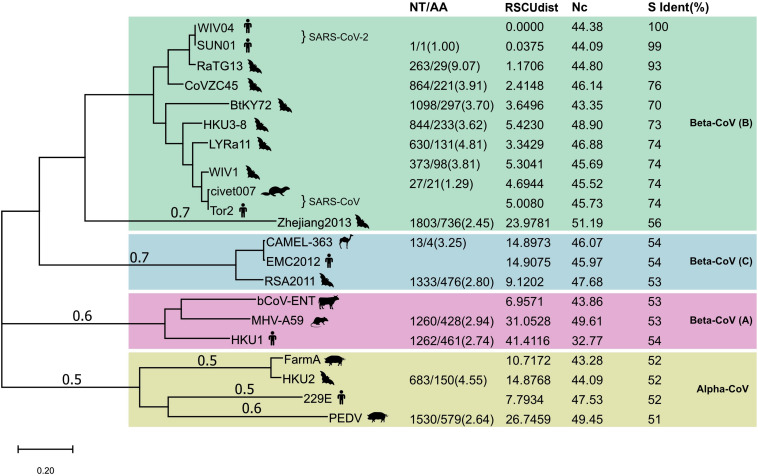
Maximum-likelihood phylogenetic tree of 21 coronavirus strains. The tree was built with the full genome and rooted against the alpha-CoV clade. NT, number of nucleotide changes; AA, number of amino acid changes. Both NT and AA were the changes from the reference strain that had no numerical value in the NT/AA column in each CoV lineage. RSCUdist, RSCU distance of the spike protein; ENc, effective number of codons; S ident (%), percent identity of the S protein sequence between different coronaviruses. The NT/AA ratio between WIV04/RaTG13 was significantly higher (chi-squared test; *p* < 0.01) than that of other strain pairs.

Next, we compared the proportion of synonymous mutations in the spike gene between WIV04 and RaTG13 or Bat-SL-CoVZC45. The ratio of nucleotide substitutions (263 NT) to amino acid substitutions (29 AA) was 9.07 from WIV04 to RaTG13, significantly higher than the ratio (3.91, 864/221) from WIV04 to Bat-SL-CoVZC45 (*p* < 0.05) (Figure 2). Similar results were observed in comparisons using their whole genomes. Consequently, the proportion of synonymous mutations (∼40%) among all currently reported SARS-CoV-2 strains is similar to that between WIV04 and Bat-SL-CoVZC45 (39.1%) but is dramatically lower than that between WIV04 and RaTG13 (90.7%).

**FIGURE 3:**
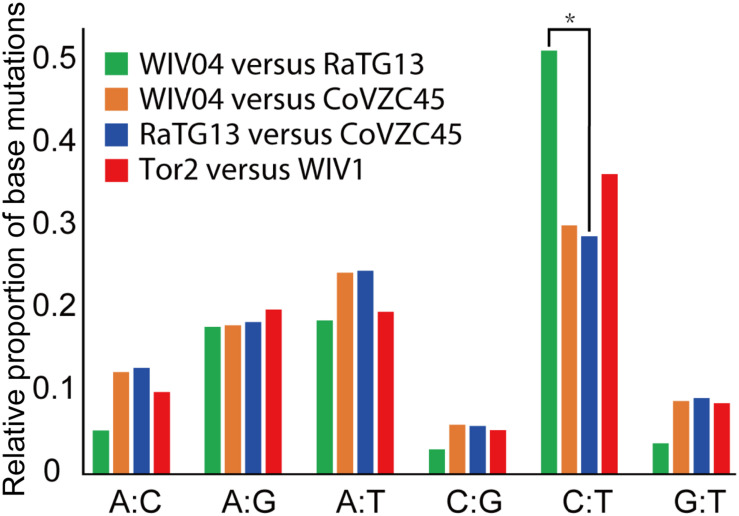
Frequency of point mutations observed in the spike gene. The frequency is the number of point mutations in each category (A:C, A:G, A:T, C:G, C:T, and G:T) divided by the total mutations. “A:C” indicates that nucleotide A was changed to C, or C changed to A. *p < 0.05.

Furthermore, the proportion of T-to-C (T:C) transitions in the whole nucleotide mutation was markedly higher between WIV04 and RaTG13 than between others (Figure 3). Interestingly, CoVs lacking a 3′-to-5′ exoribonuclease (ExoN) accumulate 15- to 20-fold more A:G and U:C transitions (Smith et al., 2013). Thus, the specific T:C transition pattern between SARS-CoV-2 and RaTG13 might have resulted from the loss function of ExoN in the process of evolution.

### The Mutation Pattern Between SARS-CoV-2 and RaTG13 Is Unique Across Coronavirus Species

To investigate whether the increased ratio of the nucleotide to amino acid changes between SARS-CoV-2 and RaTG13 is unique across all coronavirus species, we further compared their alterations in nucleotides and amino acids with other representative coronaviruses. Phylogenetic analysis of SARS-CoV-2 and its 20 closely related coronaviruses formed four well-supported clades (Figure 2). The two SARS-CoV-2 strains WIV04 (from a Wuhan patient) and SNU01 (from a Korean patient) were clustered with SARS-CoV-related strains to form clade 1, belonging to beta-coronavirus lineage B. MERS-CoV (EMC2012) from humans, CAMEL-363 from camels, and RSA2011 from bats formed clade 2, belonging to beta-coronavirus lineage C. Beta-coronavirus lineage A is formed by the representative bovine coronavirus (bCoV-ENT), murine hepatitis virus (MHV-A59), and human coronavirus HKU1. The last clade comprised four representative alpha-coronaviruses. Notably, the sequence identities between closely related representative viruses, such as WIV04 and RaTG13, Tor2 and WIV1, and SADS FarmA and HKU1, were nearly 95%.

Next, we compared the alterations in the nucleotides and amino acids between these coronaviruses. As described previously, the ratio of the number of nucleotide changes to amino acid changes (NT/AA) was as high as 9.07 when SARS-CoV-2 was compared with Bat-CoV RaTG13 (Figure 2). However, this ratio was less than 5.0 when comparing other human coronaviruses with their similar animal coronaviruses. For example, the ratio of human SARS-CoV Tor2 to bat SARS-like CoV LYRa11 was 4.81, the ratio of human MERS-CoV EMC2012 to bat SARS-like CoV RSA2011 was 2.80, and the ratio of human coronavirus 229E to bat coronavirus HKU2 was 4.55. These results indicate that the relative level of synonymous substitutions between human SARS-CoV-2 and its possible animal origin (RaTG13) is much higher than that between other human coronaviruses and their related animal strains.

### Codon Usage Is Similar Across Beta-Coronavirus Lineages

Different organisms, including different protein-coding genes of the same species, have different frequencies of codon usage (Zhou et al., 2020). The RSCU bias of coronaviruses will reveal the difference in their host source. We calculated the distance of RSCU between the spike genes of 20 representative coronaviruses and SARS-CoV-2. The codon usage difference (distance of RSCU) between SARS-CoV-2 WIV04 and Bat-CoV RaTG13 was 1.17, which was the lowest except for SNU01 (another strain of SARS-CoV-2), indicating that their codon preference was almost the same; the second lowest codon usage difference was 2.41, which was detected between SARS-CoV-2 WIV04 and Bat-SL-CoVZC45 (Figure 2). The coronaviruses in the same beta-CoV lineage (B) have a relatively close distance, except CoV Zhejiang2013. The codon usage difference of the spike gene between human coronavirus HKU1 and bovine bCoV-ENT sequence had the largest difference, with a ratio of 41.41.

Codon usage bias in a gene can be effectively measured by determining the ENc. The lower ENc values represent high codon bias with low numbers of synonymous codons used for the amino acids, and a gene with strong codon usage bias may have an ENc value less than 35. The ENc value of the WIV04 spike gene was 44.38 (Figure 2), which is similar to those of RaTG13 and other bat coronaviruses in the B, indicating that the high synonymous mutation was unlikely to be determined by codon usage bias.

### The Nucleotide Substitutions Between SARS-CoV-2 and RaTG13 Are Affected by Stronger Purifying Selection

To infer whether the retention of mutations is supported or hindered by natural selection, we further studied the non-synonymous substitution rate (dN) and synonymous substitution rate (dS) in the spike gene (Table 2). Generally, positive (Darwinian) selection increases, but negative (purifying) selection decreases the ratios of non-synonymous to synonymous substitution rates (dN/dS). Our results showed that both dN and dS of the *S* gene of SARS-CoV-2 WIV04 versus Bat-SARr-CoV RaTG13 were the lowest among all typical coronaviruses, while those of SARS-CoV Tor2 versus bat SARS-like coronavirus WIV1 were the second lowest. When the ratio of dN to dS of the spike gene is compared, all the tested dN/dS values are less than 1, indicating that these non-synonymous mutations are harmful, and negative selection will reduce their retention speed (Table 2). Among them, the dN/dS of the spike gene of SARS-CoV-2 WIV04 versus that of Bat-SARr-CoV RaTG13 was 0.04, which was the lowest among all comparisons, reconfirming that the rate of synonymous mutation was extensively high between WIV04 and RaTG13 strains. Moreover, the dN/dS rates of the polyprotein (ORF1ab) and nucleocapsid (N) genes were similar to that of the spike gene (Supplementary Table 1).

**TABLE 2:** Comparison of the evolutionary rate of the spike protein and its RBD region between different coronavirus strains.

**Coronavirus strains for pair comparison**		**Spike**			**Receptor-binding domain (RBD)**		
		**dN/Ds**	**dN**	**dS**	**dN/dS**	**dN**	**Ds**
WIV04	RaTG13*	0.04	0.014	0.31	0.1165	0.064	0.5494
WIV04	CoVZC45	0.11	0.13	1.19	0.1067	0.2213	2.0751
RaTG13	CoVZC45	0.12	0.13	1.08	0.1067	0.2213	2.0751
WIV04	Tor2	0.11	0.17	1.50	0.095	0.028	0.2945
Tor2	WIV1*	0.17	0.05	0.32	0.1592	0.1997	1.2545
FarmA	HKU2*	0.09	0.097	1.08	0.0835	0.1114	1.3342
EMC2012	RSA2011	0.21	0.29	1.38	0.4015	0.6464	1.61

*The whole genome sequence identity between these paired strains was larger than 95%.

Because the receptor-binding domain (RBD) of the spike protein is involved in interacting with human angiotensin-converting enzyme 2 (ACE2) protein, the RBD region is thought to be a preferential target of natural selection (Forni et al., 2016). Consistent with this hypothesis, our results showed that both dN and dS of the RBD region are higher than those of the whole spike gene region across all the virus pairs used in this study (Table 2). Notably, the dN/dS ratio of the RBD region in SARS-CoV-2 WIV04 was dramatically increased by approximately threefold compared with the full spike region. Consequently, these mutations might be subjected to Darwinian selection or relaxation of purifying selection.

Furthermore, MEME analysis was performed to detect positive selection on the spike gene. Significant (*p* < 0.05) pervasive episodic selection was detected in three sites (48th, 254th, and 330th positions using the reference sequence of WIV04) on the common ancestor of WIV04 and RaTG13 lineages. At the 254th position of the spike amino acid sequence, a histidine residue is present instead of a phenylalanine residue; at the 330th amino acidic position in the WIV04 sequence, a glutamine residue is present instead of a valine residue. The results described above support the action of positive selection on some sites of the spike gene during the recent evolution of SARS-CoV-2 and RaTG13.

## Discussion

In this study, we first observed that the proportion of synonymous substitutions was similar to that of non-synonymous substitutions within currently available SARS-CoV-2 strains. According to the random drift hypothesis (Castellano et al., 2018), these nucleotide differences among different SARS-CoV-2 strains may primarily result from neutral evolution. In short, no powerful factor exists to force SARS-CoV-2 to evolve in a certain direction. However, strict precautions should be taken against the strong factors that may cause directional variation of SARS-CoV-2 both in the natural environment and during infection treatment.

Second, our results showed that synonymous mutations are dramatically elevated between SARS-CoV-2 and RaTG13. The relative proportion of synonymous substitutions between human SARS-CoV-2 and its possible animal origin (RaTG13) is much higher than that between other human coronaviruses and their potential animal sources. These results indicated that the SARS-CoV-2 strains might undergo stronger purifying selection after diverging from their common ancestor. Interestingly, the nucleotide mutations were enriched in the T:C transition. The specific mutation pattern may be caused by the inactivation of RNA 3′-to-5′ ExoN (Smith et al., 2013). This increased T:C mutation implies that the ExoN of SARS-CoV-2 may be deactivated compared with that of RaTG13. Moreover, RNA mutagen 5-fluorouracil (5-FU) treatment can increase U:C and A:G transitions as well (Smith et al., 2013). Therefore, the underlying mechanisms of such potential mutations between SARS-CoV-2 and RaTG13 require further investigation in the future.

Previous studies on codon usage bias between viruses and their hosts have suggested that viruses tend to evolve codon usage bias comparable to their hosts (Bahir et al., 2009). Generally, RNA viruses usually comprise high codon usage bias, which helps in replication and host adaption with preferred codons. However, our results showed that codon usage was similar between SARS-CoV-2 and other strains in beta-coronavirus lineage B, suggesting that the presumptive mutation pattern was not determined by codon usage bias.

The non-synonymous (dN) to synonymous substitutions (dS) ratio in protein-coding genes is commonly used to detect the selection pressure during gene evolution. A dN/dS ratio larger than 1 indicates positive selection, while a dN/dS ratio less than 1 indicates negative selection acting on protein-coding genes. Our results showed that the dN/dS between SARS-CoV-2 and RaTG13 was less than 0.1 and significantly lower than that of other paired strain comparisons. These results indicate that the SARS-CoV-2 virus exhibits extraordinarily stringent negative selection pressure if it evolved from RaTG13. By contrast, the relatively high dN/dS ratio in the RBD region of the spike protein suggests that the selective pressure acting on this region is relaxed, and some sites may be undergoing positive selection. This increased evolutionary rate can be explained by the important function of the spike protein, which participates in host-specific recognition and undergoes several drastic changes during virus infection. For example, its large parts are cleaved during infection by cellular proteases, exposing the receptors to activate viral attachment to the host (Lu et al., 2015). However, this result should be addressed carefully because the RBD region of the spike gene from SARS-CoV-2 is divergent from that of RaTG13, suggesting that it may have originated from homologous recombination between RaTG13 and one yet-unknown coronavirus (Zhang C. et al., 2020).

In summary, through comprehensive comparative analysis between SARS-CoV-2 and other coronaviruses, we found that synonymous mutations were dramatically elevated between SARS-CoV-2 and RaTG13 compared with other coronavirus strains, and nucleotide mutations were enriched in the T:C transition. Because SARS-CoV-2 is supposed to originate from Bat-CoV RaTG13, the increased synonymous substitution between SARS-CoV-2 and the RaTG13 strain suggests that the SARS-CoV-2 genome should be under stringent negative (purifying) selection. Moreover, the mechanism underpinning the increased T:C mutations requires further investigation.

## Data Availability Statement

The original contributions presented in the study are included in the article/Supplementary Material, further inquiries can be directed to the corresponding author/s.

## Author Contributions

YL conceived and designed the study. JC and XL completed the data collection. LL and GL conducted statistics and analysis of the data. All authors contributed to the article and approved the submitted version.

## Conflict of Interest

The authors declare that the research was conducted in the absence of any commercial or financial relationships that could be construed as a potential conflict of interest.

## Footnotes

1 http://www.datamonkey.org/

2 http://codonw.sourceforge.net/

3 http://www.kazusa.or.jp/codon/
