# Supplementary material for: Comparative Genomic Analyses Reveal a Specific Mutation Pattern Between Human Coronavirus SARS-CoV-2 and Bat-CoV RaTG13
Source: Front Microbiol. 2020 Nov 30;11:584717. doi: 10.3389/fmicb.2020.584717 (PMC7793997; doi:10.3389/fmicb.2020.584717)
Supplement: Supplementary file 1 [file Table_1.docx]

**Supplementary material**

**Supplementary Table 1. Comparison of the evolutionary rate of ORF1ab (P) and nucleocapsid (N) gene between different coronavirus strains**

| Coronavirus strains for pair comparison | | P | | | N | | |
| --- | --- | --- | --- | --- | --- | --- | --- |
|  |  | dN/dS | dN | dS | dN/dS | dN | dS |
| WIV04 | RaTG13* | 0.0428 | 0.0052 | 0.1226 | 0.0453 | 0.0065 | 0.1433 |
| WIV04 | CoVZC45 | 0.1098 | 0.0354 | 0.3222 | 0.0494 | 0.0268 | 0.5422 |
| RaTG13 | CoVZC45 | 0.1109 | 0.0342 | 0.3085 | 0.0506 | 0.028 | 0.5544 |
| WIV04 | Tor2 | 0.0172 | 0.001 | 0.0607 | 0.0589 | 0.0077 | 0.1306 |
| Tor2 | WIV1* | 0.1458 | 0.0557 | 0.3819 | 0.0898 | 0.0968 | 1.078 |
| FarmA | HKU2* | 0.0929 | 0.025 | 0.2689 | 0.9776 | 0.3599 | 0.3682 |
| EMC2012 | RSA2011 | 0.1088 | 0.0542 | 0.4983 | 0.0785 | 0.0406 | 0.5167 |

*The whole genome sequence identity between these paired strains was larger than 95%.
